# Neuroimmunomodulation in the Gut: Focus on Inflammatory Bowel Disease

**DOI:** 10.1155/2016/1363818

**Published:** 2016-07-04

**Authors:** Claudio Bernardazzi, Beatriz Pêgo, Heitor Siffert P. de Souza

**Affiliations:** ^1^Serviço de Gastroenterologia & Laboratório Multidisciplinar de Pesquisa, Hospital Universitário, Universidade Federal do Rio de Janeiro, 21941-913 Rio de Janeiro, RJ, Brazil; ^2^D'Or Institute for Research and Education (IDOR), 22281-100 Rio de Janeiro, RJ, Brazil

## Abstract

Intestinal immunity is finely regulated by several concomitant and overlapping mechanisms, in order to efficiently sense external stimuli and mount an adequate response of either tolerance or defense. In this context, a complex interplay between immune and nonimmune cells is responsible for the maintenance of normal homeostasis. However, in certain conditions, the disruption of such an intricate network may result in intestinal inflammation, including inflammatory bowel disease (IBD). IBD is believed to result from a combination of genetic and environmental factors acting in concert with an inappropriate immune response, which in turn interacts with nonimmune cells, including nervous system components. Currently, evidence shows that the interaction between the immune and the nervous system is bidirectional and plays a critical role in the regulation of intestinal inflammation. Recently, the maintenance of intestinal homeostasis has been shown to be under the reciprocal control of the microbiota by immune mechanisms, whereas intestinal microorganisms can modulate mucosal immunity. Therefore, in addition to presenting the mechanisms underlying the interaction between immune and nervous systems in the gut, here we discuss the role of the microbiota also in the regulation of neuroimmune crosstalk involved in intestinal homeostasis and inflammation, with potential implications to IBD pathogenesis.

## 1. Introduction

The enteric nervous system (ENS) constitutes a major autonomic division of the nervous system that provides the intrinsic innervation of the gut, capable of controlling different functions, such as motility, mucosal secretion and absorption, mucosal growth, local blood flow, and the immune function [1]. The ENS can be influenced by the central nervous system (CNS), establishing a two-way relationship treaded by the brain-gut axis. Actually, all basic gastrointestinal (GI) functions can be regulated by the ENS, but the coordination of the gut function and the maintenance of the homeostasis of the organism both require a communication between the GI tract and the CNS [2]. The ENS consists of two interconnected networks of ganglia and fibers encircling the GI tract, arranged in a peculiar way, which confers the ability to mediate its own reflexes. In this context, evidence shows that ENS can work independently of the CNS [3]. Because of these properties, ENS is a primary regulator of GI functions and has been referred to as a second brain in humans [3]. However, despite the ability of the ENS to regulate motility and secretion in an autonomous way, there are many connective links with the CNS, working in a bidirectional fashion [4].

Nerve cells located within the mucosa are in close proximity to immune cells, where they produce and respond to several common mediators [5, 6]. Upon ENS activation, mucosal immune cells expressing receptors for neurotransmitters can be stimulated to migrate, degranulate, differentiate, or secrete immunoglobulins, for example, [7–9]. Therefore, the communication between the ENS and the immune system within the mucosa participates in the control of major GI functions but can also be associated with pathological conditions, such as inflammatory bowel disease (IBD).

## 2. Enteric Nervous System

The ENS, commonly called “the little brain of the gut,” is a constituent of the peripheral nervous system which is composed by an intrinsic network containing enteric neurons cell bodies, intestinal cells of Cajal (ICC), interneurons and motor neurons, and enteric glial cells (EGC). The latter have been extensively studied in recent years and consist of small cells with stellate shape that is associated with neuron cell bodies and nerve fibers in intraganglionic connections. Evidence supports that EGC are very similar to astrocytes, not only morphologically, but also functionally [10]. All these elements are grouped into ganglia and interconnected by bundles of nerve processes forming plexuses, including the myenteric (Auerbach's) and submucosal (Meissner's) plexus.

**Figure 1:**
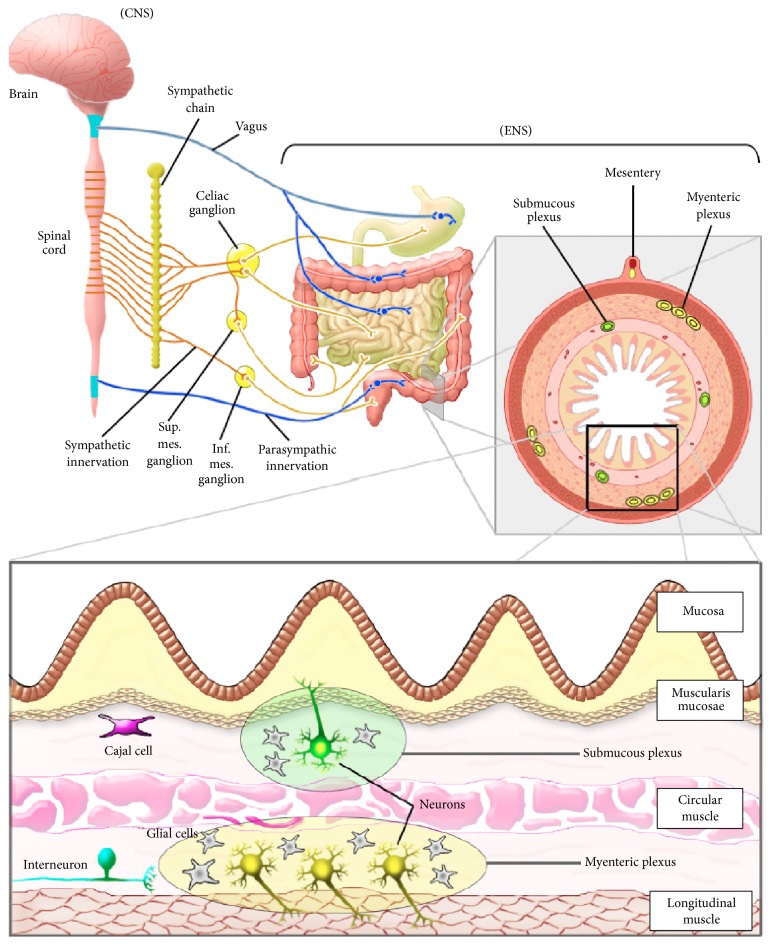
Schematic illustration showing the interaction between the central nervous system (CNS) and the enteric nervous system (ENS). The sympathetic and parasympathetic innervations interact with neurons in the gastrointestinal tract, passing the sympathetic innervations first through the celiac ganglion and the superior mesenteric ganglion (sup. mes. ganglion) and the inferior mesenteric ganglion (inf. mes. ganglion). The quadrant below the CNS and the ENS represents the intestinal mucosa and its myenteric and submucosal plexuses.

The myenteric plexus extends from the upper esophageal to the external anal sphincter, situated between the longitudinal and the circular muscle layer. The submucosal plexus is restricted to the submucosa of the small and large intestines. In human, for instance, two ganglionated submucosal plexuses can be distinguished. Thereby, there is an internal submucosal plexus, which lies in the inner half of the submucosa, and also an external submucosal plexus, this one situated close to the inner border of the circular muscle layer [11] (Figure 1).

In the present review, the current knowledge and the clinical implication of ENS in IBD will be discussed.

## 3. Mediators of the Enteric Neurons System

Neurotransmitters are molecules produced by neurons that play a role in the transmission of information cell to cell, in maintaining stimulus of impulses, and act in the neuromuscular junction. When an action potential reaches the terminal button of a presynaptic neuron, a voltage-dependent calcium channel opens, resulting in the release of neurotransmitters in the synaptic cleft [12, 13]. These molecules, also known as neuropeptides, including acetylcholine, serotonin, substance P, corticotropin-releasing hormone, and vasoactive intestinal peptide (VIP), are distributed throughout the gut and participate in normal homeostasis as well as in inflammatory processes [14].

Acetylcholine (ACh) plays a role in both CNS and in ENS upon ligation with ACh receptors, ligand-dependent cation channels, of which the two major classes are the muscarinic and the nicotinic receptors [15, 16]. The activation of ACh receptors by binding to ACh determines the depolarization of the postsynaptic neuron and the initiation of a new action potential [17, 18].

Another neurotransmitter, abundant in intestinal neuroendocrine cells, is serotonin (5-hydroxytryptamine, 5-HT). About 95% of 5-HT in the human body is found in GI tract and its action influences luminal contents and secretions [19]. The most frequent component of the enteroendocrine cell population is the enterochromaffin cell (EC), estimated to contain 90% of the total intestinal 5-HT, while 10% is in enteric neurons [19–21]. Once released basolaterally, 5-HT can perform multiple functions, including action on primary intrinsic afferent neurons initiating peristaltic reflex, the stimulation of cholinergic neurons to release acetylcholine, resulting in smooth muscle contraction, and the stimulation of inhibitory nitrergic neurons to release NO, which results in smooth muscle relaxation [22]. In addition, 5-HT participates in potential mucosal protecting processes, stimulating active ion, mucus, and fluid secretion. The secretory effect of 5-HT is mediated by epithelial 5-HT2 receptors and neuronal 5-HT1P, 5-HT3, and 5-HT4 receptors [22]. In the GI tract, the abnormal secretion of 5-HT has been associated with various effects, such as nausea, vomiting, and alterations in the intestinal secretion and peristalsis [19], indicating that this neuroendocrine transmitter plays an important role in the regulation of gastrointestinal functions. Interestingly, the major source of 5-HT, EC cells, also expresses toll-like receptors, which make them capable of sensing microorganisms [23, 24].

Substance P (SP) is localized in enteric nerves distributed throughout the gut and present in myenteric and submucosal plexuses [25, 26]. The effects attributed to SP, such as regulation of mucosal permeability [27], motility [28], secretion [29], epithelial cell proliferation [30], and inflammation [31], are initiated upon ligation with G-protein-coupled NK-1R, which is present in both the small and large bowel of animals and humans [32, 33]. In regard to intestinal inflammation, SP-NK-1R-induced proinflammatory signaling was shown to result in the production of a downstream cascade of proinflammatory molecules mediated by the activation of NF-kappa B [34, 35], or p38 mitogen-activated protein kinase [36]. It is interesting to note that NF-kappa B can also modulate NK-1R expression [37]. SP has been identified also in immune cells, such as dendritic cells, mononuclear phagocytes, and lymphocytes [28, 38–41], while NK-1R can be present in T and B cells, macrophages, dendritic cells, neutrophils, natural killer cells, and eosinophils [28, 39, 42–45]. Moreover, SP and NK-1R can promote inflammation by regulating intestinal angiogenesis through the increase in the expression of CCN1 (CYR61) [46], which is upregulated in the colon from UC patients [47].

Nitric oxide (NO) is regarded as a cellular signaling molecule, which can play different roles in the GI tract, such as participating in the maintenance of mucosal integrity and also regulating vascular tone and the mucosal blood flow [48]. NO is catalyzed by one of the isoforms of nitric oxide synthase (NOS), of which the GI tract expresses constitutively two: endothelial NOS (eNOS) and neuronal NOS (nNOS) [49]. An additional isoform is the inducible isoform (iNOS), which is upregulated in response to inflammation and other stimuli. The increase in NO concentration, in turn, results in the production of reactive oxygen species and consequently also potential oxidative stress [50]. In fact, increases in NO concentration have been associated with harmful effects in the GI tract, including human IBD and also experimental colitis [51]. Interestingly, the increase in both iNOS expression and NO production in patients with ulcerative colitis was shown to be secondary to enteroglial-derived S100B protein upregulation. This information unveils an unexpected mechanism by which the enteric glia mediates mucosal NO-dependent inflammatory responses [52]. The role of NO in intestinal homeostasis and inflammation is further reinforced by the fact that VIP is released from nerve terminals containing NOS. Such peptides are thought to comprise a nonadrenergic and noncholinergic nerve transmission circuit within the gut [53, 54].

## 4. Enteric Nervous System in the Pathogenesis of Inflammatory Bowel Disease

During the course of IBD, the marked immune cell infiltration and the activation of mechanisms that modulate cell turnover within the intestinal epithelium can lead to permeability changes with potential disruption of the intestinal barrier [55–57]. This milieu of changes, which in certain levels contributes to the pathogenesis of IBD, can further progress and ultimately affect also the morphology and function of the ENS [58]. Abnormalities such as axonal rupture of nerve fibers, damage of neuronal cell bodies, hyperplasia of EGC, and increase of axonal necrosis of gut nerves have been associated with IBD [59–62]. Villanacci et al., for example, observed differences in the number of neuronal cell bodies, ICC and EGC in patients with Crohn's disease (CD) and ulcerative colitis (UC). Interestingly, abnormalities such as a reduction of enteroglia, found in noninflamed areas of the intestine, led to the hypothesis of a pathogenic role of the ENS in IBD [63]. Ohlsson et al. corroborated these findings and further demonstrated the presence of visceral ganglioneuritis, in addition to atrophy and vacuolar degeneration of ICCs in the small bowel of patients with CD [64]. On the other hand, in another study investigating the noninflamed tissue of CD patients, the transmitter colocalization patterns in rectal submucosal neurons by immunohistochemistry revealed an increase in the vasoactive intestinal polypeptide (VIP) population, extensive colocalization of choline acetyltransferase (ChAT) and NOS, and hypertrophied calcitonin gene-related peptide (CGRP) fibers [65], supporting the occurrence of adaptive alterations in the ENS in CD.

In the context of experimental colitis, enteric nervous abnormalities have also been reported in animal models. For example, results from a study using trinitrobenzene sulfonic acid- (TNBS-) induced colitis have shown that, in the beginning of inflammation, 20% of myenteric neurons are rapidly lost [66], and neuronal loss has been attributed to cell death induced by the inflammatory process consequent to TNBS-induced colitis and associated with infiltration of neutrophils [66, 67]. Hence, it is probable that the activation of immune cell-related molecules involved in IBD pathogenesis also might be responsible for the abnormalities of ENS.

## 5. Immune Cells Interaction with ENS

The presence of neuropeptides and neurotransmitter receptors on immune cells represents a strong indication of the integration between the ENS and the immune system.

### 5.1. T Cells

In normal conditions, mucosal T cells respond to different environmental challenges orchestrating the immune response in an adaptable fashion [68]. In IBD, such plasticity of T cells appears to be compromised, resulting in chronic inflammation [69]. Currently, in CD, the immune response has been regarded as a mixture of a T helper type 1 (Th1) and Th1/Th17 phenotypes [70], while in UC it comprises an atypical Th2 phenotype, with the addition of Th9 [71], and a less prominent Th17 response [72]. Recently, complex modulatory mechanisms reciprocally involving the ENS and the mucosal immune system have been recognized [73]. For instance, the vagus nerve appears to play an important role in this integrative process [74, 75] and, when stimulated, it acts as an anti-inflammatory promoter activating sympathetic neurons in the mesenteric ganglion that release noradrenalin, which activates T cells. These T cells, defined as memory cells, in turn, release acetylcholine (Ach) that inhibits proinflammatory cytokines from macrophages [76]. Particularly, in physiological conditions, T cells have been seldom seen in the proximity to ENS. Nevertheless, Sayani et al. demonstrated that, in the context of experimental intestinal inflammation, mucosal T cells increased, but being consistently excluded from ganglia. Such effect has been attributed to the expression of Fas-ligand (Fas-L) on enteric neurons, which appears to protect them against Fas-Fas-L-induced apoptosis, possibly further contributing to the resolution of inflammation [77]. Taken together, these evidences support the idea of a neural information system capable of controlling innate and adaptive immune responses.

**Figure 2:**
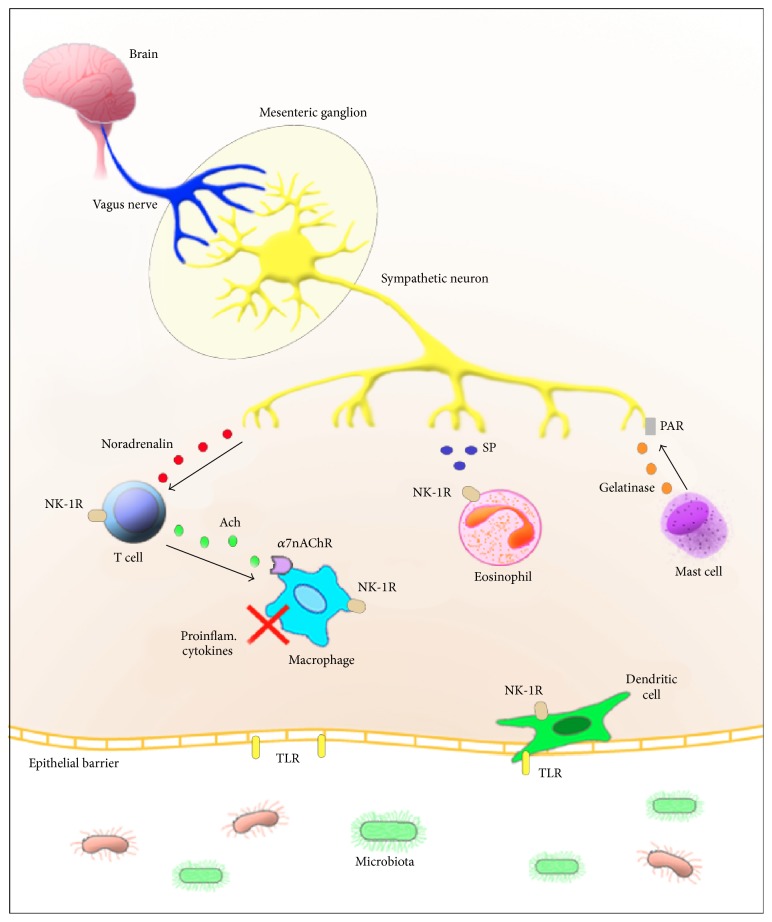
Interaction between the enteric neuron system (ENS) and mucosal immune cells. Upon vagus nerve stimulation, the sympathetic innervation secretes neurotransmitters that can modulate immune cells and the inflammatory response. The intestinal microbiota also participates in the inflammatory response fine-tuning the interaction between the ENS and mucosal immune cells.

Another interesting example of this integration is the evidence of hypothalamic-pituitary-adrenal axis regulation of intestinal inflammation [78], through the anti-inflammatory effects of glucocorticoids [79]. In IBD, the activated inflammatory cascade has been shown to affect GI motility and function [80], providing another indication of an intimate communication between ENS and the mucosal immune system (Figure 2).

### 5.2. Macrophages

In the intestinal mucosa, resident macrophages are usually present in the lamina propria where they preferentially locate in the subepithelial area to constitute the first line of defense against potentially harmful external stimuli [81]. Nevertheless, macrophage subsets are distributed also below the epithelial layer, towards the submucosa and muscularis externa, exhibiting distinct phenotypes and probably specific functions [82].

In the gut, vagus nerve stimulation has anti-inflammatory properties, also known as cholinergic anti-inflammatory pathway, with influence on diverse immune-mediated disorders [83]. For example, vagus nerve activation by electrical stimulation and systemic nicotinic receptor agonists was shown to abate intestinal inflammation, by reducing the production of proinflammatory cytokines by macrophages [84]. Recently, the interaction between vagal efferents and intestinal macrophages has been investigated, but no clear evidence of direct modulation could be demonstrated. Hence, researchers proposed that vagal modulation of intestinal macrophages could actually be indirect, probably via cholinergic and nitrergic/VIPergic enteric neurons [85].

TNF-alpha, a prototypical Th-1-type of proinflammatory cytokine, associated with IBD and particularly with CD [86], can be modulated by the vagus nerve, through the inhibition of macrophage release. Notably, Wang et al. reported that the nicotinic acetylcholine receptor alpha 7 subunit (*α*7nAChR) is essential in acetylcholine inhibition of TNF-alpha production by macrophages [87], revealing a mechanism of neuromodulation of the immune response. In another study on the subject, the anti-inflammatory action of the vagus nerve in the intestine was shown to be dependent on its interaction with cholinergic myenteric neurons in intimate association with the muscularis macrophages. In addition, it has been suggested that resident macrophages expressing *α*7nAChR would probably be the ultimate intestinal target of such anti-inflammatory pathway [88].

### 5.3. Neutrophils

 Like T cells, neutrophils are rarely observed in submucosal and myenteric plexuses in normal conditions, but during chronic active IBD these inflammatory cells accumulate and infiltrate the mucosa, contributing to the tissue injury [89] and possibly also affecting the ENS.

In experimental colitis in rats, induced by dinitrobenzene sulfonic acid (DNBS) administration, Sanovic et al. demonstrated a significant neuronal reduction in the inflamed segments in the first 24 hours, with less than half of neurons remaining by days 4 to 6 and thereafter, when inflammation had diminished. The neuronal damage was associated with the early accumulation of neutrophils and eosinophils within the ganglia, an effect more prominent in the submucosal ganglia [90]. In another experimental study with DNBS-induced colitis, mice treated with anti-neutrophil antibody had a significant attenuation of tissue damage and a greater number of neurons compared to nontreated colitic mice [67]. In conjunction, these data suggest that neutrophils might participate in the loss of ENS neurons during inflammatory intestinal conditions, including IBD.

### 5.4. Eosinophils

In the past three decades, relevant evidences point to an important role of eosinophils in IBD [91–94]. Smyth et al., studying different clinical stages of IBD, demonstrated that the major basic protein (MBP, a cationic protein released by eosinophils, which can be cytotoxic in high concentrations), as well as eosinophils, localizes to nerves and ganglia in the mucosa of patients with refractory disease [95]. Moreover, an increased expression of eotaxin-3 and ICAM-1, molecules involved in tissue eosinophilia and leukocytes transmigration, respectively, was detected in the same mucosal location, in the vicinity of nerves and ganglia [96]. In refractory CD patients, eosinophils have been found close to nerves within the smooth muscle layer. Furthermore, eosinophils localized specifically to SP and ChAT nerves, in CD mucosa, suggesting an indirect role for eosinophils also as mediators of smooth muscle contraction and gut motility [95]. Eosinophils have also been implicated in the pathophysiology of UC, and the involvement in mucosal inflammation and destruction was suggested to be associated with SP innervation and the neurokinin-1 receptor (NK-1R) expression, most marked in areas of mucosal accumulation of eosinophils [97, 98].

### 5.5. Mast Cells

Mast cells play an important role in innate and adaptive immune responses by regulating the allergic reaction and defense against pathogens. Their growth and proliferation are regulated by cKit ligand stem cell factor (SCF), nerve growth factor (NGF), IL-3, IL-4, IL-9, and IL-10 [99]. Under physiological conditions, mast cells are present in the mucosa, submucosa, and the circular muscle layer [100] and play a role in allergic diseases by releasing proteases, cytokines, and chemokines [101].

Increased mast cell numbers are observed in the proximity of mucosal enteric nerve fibers in a model of visceral hypersensitivity in rats [102]. In a model of food allergy in mice, Lee et al. observed that nerve fibers expressing the neurotransmitter CGRP (calcitonin gene-related peptide) were increased and colocalized to mucosal mast cells in the colonic mucosa [103]. The proximity of mast cells to mucosal enteric nerve fibers has also been described in adult patients with irritable bowel syndrome (IBS) [104], a disease in which clinical manifestations notably overlap with IBD [105]. In an animal model of IBS, for instance, Barbara et al. showed that mucosal mast cells are capable of exciting nociceptive visceral sensory nerves, suggesting their implication in visceral hypersensitivity in IBS [106]. Ileal segments of patients with CD were shown to host a marked number of mast cells displaying piecemeal degranulation associated with ICC, in the muscularis. Of note, various types of injury were described in ICC, probably due to direct contact with mast cells and the chronic release of their potentially cytotoxic granule contents [107].

## 6. Purinergic Receptors

Purinergic receptors, also known as purinoceptors, are transmembrane receptors including the P1 and P2 subtypes. P2 is composed of two subforms, namely, P2Y and P2X. P2Y, and its variants, is a G-protein coupled receptor, while P2X, and its variants, is a ligand gate ion channel [108]. P2X7 is the most studied purinoceptor, and it has been implicated in the induction of caspase activity, cytokine secretion, and cell death. The ligand for P2X7 is adenosine triphosphate (ATP), a molecule that at high concentrations functions as a danger signal associated with tissue inflammation and damage [108], therefore constituting a damage-associated molecular pattern (DAMP). Gulbransen et al. studied the activation of enteric neuronal P2X7 receptor during inflammation in animal models and demonstrated that the myenteric neuronal density decreased during colitis, but with a pretreatment with oxidized ATP (an antagonist of P2X7 receptor) there was a protection against inflammation-induced neurons loss. On the other hand, using BzATP, an agonist of P2X7 receptor, neural packing density was reduced [109]. This phenomenon was also observed when ChAT-, calbindin-, calretinin-, anti-HuC/D-, and NOS-neurons, in which cells express P2X7 receptor, were decreased during colitis [110]. Gulbransen et al. also demonstrated that, in addition to the P2X7 receptor expression in myenteric neurons, these cells also express Panx-1, absent in EGC [109]. The P2X7 receptor expression has been associated with cell death during intestinal inflammation in human and experimental IBD [111], and the activation of P2X7 receptor-Panx-1 was proposed to contribute to neuron death by activation of a complex of caspases. In this regard, the release of ATP by Panx-1 also mediated death of EGC by phospholipase-C (PLC) pathway initiated by P2Y1 [109]. Furthermore, it has been shown that the rapid loss of myenteric neurons involves not only caspase-dependent pathway but also other multiprotein complexes, such as the inflammasome [109]. In accordance with this, blocking either P2X7 receptor or Panx-1, associated with inflammasome triggering, was shown to prevent neurons death [109]. Other purinergic receptors have been described within enteric neurons. In the myenteric and submucosal plexuses, the ChAT, calbindin, calretinin, and NOS neurons also express P2X2 receptor, which can bind to ATP and mediate synaptic transmission [112]. In the myenteric plexus, the P2X6 receptor is expressed in neurons that resemble Dogiel type II neurons [113]. The G-protein coupled purinergic receptor P2Y2 is distributed in both plexuses, in neurons and fibers. In the myenteric plexus of small intestine of guinea pig, P2Y2 receptor is associated with neuropeptide-Y and calretinin [114]. In a model of chronic inflammatory pain, this purinergic receptor is increased in peripheral cutaneous sensory neurons that innervate injured tissue [115]. Recently, it has been demonstrated that ATP liberated from the gut epithelium during cell stressful stimuli, can mediate excitation of visceral afferents through P2X receptors [116], and also stimulate mouse and human visceral nociceptors through P2Y receptors [117]. These findings appear to implicate ATP in the generation of functional GI alterations, as a neurogenic component of the inflammatory process. In addition, ATP, regarded as DAMP, triggers mechanisms downstream of P2X7 and mediates the inflammasome activation, probably contributing to the maintenance and amplification of the inflammatory response [118, 119].

## 7. Enteric Glial Cells

Under physiologic conditions, intestinal barrier is relatively impermeable, but during pathologic conditions, barrier disruption has been associated with the development of GI diseases, including inflammatory disorders [120]. Regarding the intestinal barrier, results of recent studies suggest an important contribution of EGC in the maintenance of normal functions. EGC are abundant in GI tract [121, 122] and are in close proximity to the intestinal epithelial border and in contact with epithelial basement membrane [121].

The genetic ablation of enteric glial cells using transgenic mice expressing herpes simplex virus thymidine kinase from the mouse glial fibrillary acidic protein (GFAP) promoter, performed by Bush et al., showed that when the animals were treated with ganciclovir, an increased inflammatory response ensued and led to death with an underlying severe jejunoileitis [123]. In another study using the same experimental model, Savidge et al. demonstrated that the involvement of EGC with the intestinal barrier function could, at least in part, be due to the release of S-nitrosoglutathione (GSNO), a small molecule that regulates the tight junctions of the epithelial cells. Of notice, GSNO was also shown to be able to restore mucosal barrier function in CD colonic mucosal specimens [124]. In another experimental study focused on EGC, Zhang et al. revealed that glial-derived neurotrophic factor (GDNF), another molecule released by EGC, could also regulate the integrity of the intestinal barrier. Using the dextran sodium sulfate- (DSS-) induced colitis model, the investigators prevented the increase in intestinal permeability and the full inflammatory response, by treating animals with GDNF [125]. Subsequently, another study proposed the existence of an EGC self-protecting mechanism, in which GDNF protects EGC from apoptosis. The authors hypothesized that alterations in such autocrine loop would lead to a defective barrier, mucosal disruption, and development and enhancement of CD inflammation [126]. It is intriguing to note that, in the noninflamed intestinal mucosa of patients with CD, the EGC network was particularly disrupted [127] corroborating the idea that the loss or decrease of EGC might contribute to the pathogenesis of IBD.

## 8. Involvement of Intestinal Microbiota in the ENS

Trillions of bacteria colonize the gut, with hundreds of different species unevenly distributed throughout the GI tract, basically shaped by diet and immune and genetic factors of the host [128]. In humans, chronic inflammatory disorders have been associated with abnormalities in the microbiota, which may actively modulate disease phenotypes and behavior [129, 130]. In CD, for example, it has been widely accepted that abnormalities of the gut microbiota are present, where there is either an altered composition of the microbiota or an abnormal immune response against the commensal microbiota, or both [131]. Another study in CD demonstrated an increased abundance in Bacteroidetes and Proteobacteria in contrast to a decrease in Firmicutes [132] and probably more importantly a reduction in bacterial diversity [133]. In UC, there is still limited evidence for a major pathogenic role of the microbiota, but dysbiosis has also been reported [134].

In virtue of the potential ability of microorganisms to deregulate the physiological equilibrium, innate immune system is the first to respond to microbiota antigens through the recognition of microbial associated molecular patterns (MAMPs) at the transmembrane or cytosolic receptors, known as pattern recognition receptors (PRRs) [135, 136]. These receptors comprise three distinct families: toll-like receptors (TLRs), the nucleotide oligomerization domain- (NOD-) like receptors (NLRs), and retinoic acid inducible gene I- (RIG-I-) like receptors (NLRs). The best-characterized PRR in mammals is the TLRs family [137]. The TLRs are transmembrane proteins that can be expressed in different sites of the gut [138, 139] and also in components of the nervous system [140]. They trigger the activation of nuclear factor kappa-B (NFkB) through MyD88 and other intracellular mediators, leading to the production of proinflammatory cytokines (as reviewed by Elia et al. [135]). In humans, the enhanced expression of TLR-2 and TLR-4 by crypt epithelial cells demonstrated in active IBD was hypothesized as an indication of a greater ability to respond to distinct bacterial products [141].

Using animal models, Brun et al. found that the absence of TLR2 determines changes in the architecture of the myenteric and submucosal plexuses, leading to bowel dysmotility and increased susceptibility to intestinal inflammation [142]. In another study analyzing the influence of gut microbiota on the ENS, dysbiosis induced by antibiotics led to local changes in the innate immune system including TLRs and in the expression of sensory-related systems in mice [143]. Of note, results from another experimental study have shown that the exposure of the intestinal interstitium to bacterial cell products can activate nociceptive dorsal root ganglion neurons, leading to production of inflammatory cytokines and increased excitability, directly or independent of TLR signaling [144].

The colonization of the gut by microorganisms starts early in life and is critically important in several functions of the normal GI physiology [145, 146] and also the maturation of the mucosal immune system [147]. For example, in a study with germ-free mice investigating the electrophysiological properties of neurons in the myenteric plexus of the ENS, commensal microbiota was shown to be necessary for normal excitability of gut sensory neurons [148]. Further studies on the subject confirmed that germ-free mice exhibit less excitable intrinsic primary afferent neurons [149], which can be enhanced by the exposure to polysaccharide A [150]. An additional study using germ-free mice corroborated previous findings, demonstrating that the microbiome is crucial for both intrinsic and extrinsic nerve function and gut-brain signaling [151]. Recently, Collins et al. investigated whether the microbiota could influence the postnatal development of the ENS. Investigators found that germ-free mice have a decrease in nerve density and fewer neuronal cell bodies in myenteric ganglia, while in the small bowel, an increased proportion of inhibitory nitrergic neurons was detected. These results appear to support the hypothesis that early exposure to luminal microorganisms is pivotal for the postnatal development of the ENS [152].

Another set of important receptors able to interact with the ENS and modulate neurally mediated intestinal functions is the proteinase-activated receptors (PARs), expressed in different cell types in the gut. PARs belong to a group of G protein-coupled receptors that are activated by proteolytic cleavage [153, 154]. Both PAR1 and PAR2 have been shown to be functional in ENS cells. For instance, it has been demonstrated that PAR2 activation results in depolarization and increased number of action potentials in myenteric [155] and in submucosal neurons [156]. Moreover, PAR1 and PAR2 agonists induce calcium mobilization in myenteric [157] and dorsal root ganglia [158] neurons. In addition to the association with intestinal hyperalgesia and hypersensitivity [159], PAR2 has been shown to actively participate in neurogenic inflammation in the mouse colon [160]. Regarding the potential interaction with the ENS, the release of proteases from activated mast cells, for example, was shown to cleave PAR2 on submucosal neurons, determining acute and long-term hyperexcitability [161] (Figure 2). Interestingly, a recent study has demonstrated that secreted* E. faecalis* proteins, namely, gelatinase, induced permeability in the colonic epithelia of mice, which was absent in PAR2-deficient animals [162]. Together, these results strongly suggest that bacterial enzymes can regulate enteric epithelial permeability and neurogenic inflammation via intestinal PAR. The main findings of PAR receptors are exposed in Table 1. These evidences indicate the existence of interactions between the gut microbiota and the host, with effects on the ENS.

Finally, the accumulating evidence of multidirectional signaling involving the multiple components of the gastrointestinal system, including the bidirectional interplay by which the nervous system modulates the immune response, suggests that these neuronal circuits and neuromediators could be used for novel therapeutic strategies. In addition to gastrointestinal motility, sensitivity, and pain, such therapeutic approach should also provide the possibility of reestablishing immune tolerance and effective controlling chronic intestinal inflammation, for example, through the activation of the vagal anti-inflammatory pathway or the development of new pharmacological agents to control the afferent neuronal signaling.

## 9. Conclusion

The CNS interacts dynamically with the immune system to modulate inflammation through humoral and neural pathways. Neuroimmune interactions within ENS can modulate gut functions, such as motility, ion transport, and mucosal permeability, contributing to the pathophysiology of several intestinal diseases, including IBD. Intestinal inflammation has been implicated in neuroplasticity, degeneration of the ENS, and alterations in the enteric glia, with an important contribution attributed to oxidative stress. The microbiota also plays a critical role in the intestinal homeostasis and neurogenic inflammation, as it drives postnatal development of the ENS, and affects the intrinsic and extrinsic nerve function and gut-brain signaling. Further investigation of these counterregulatory mechanisms will provide additional insights into neuroimmunomodulation, potentially leading to the identification of novel therapeutic targets for the treatment of inflammatory bowel disorders.

## Figures and Tables

**Table 1:** Main findings of PAR receptors.

Studies	PARs	Samples	Methods	Results
Corvera et al. (1999) [157]	PAR1 & PAR2	Small intestine of guinea pig	Primary culture RT-PCR	PAR1 and PAR2 are expressed in myenteric neurons that express excitatory and inhibitory neurotransmitters and purinoceptors

Green et al. (2000) [163]	PAR2	Porcine ileum	Immunohistochemistry	Cholinergic and noncholinergic submucosal neurons

Buresi et al. (2005) [164]	PAR1	Mouse colon	RT-PCR Immunohistochemistry	Expressed in full-thickness specimens and mucosal scraping of colon Localized on epithelial cells and on neurons in submucosal ganglia

Ikehara et al. (2010) [165]	PAR1 & PAR2	Mouse cecum	Electrical measurements	PAR1-mediated Cl^−^ secretion might occur by activation of the receptor on the submucosal secretomotor neurons; PAR2-mediated Cl^−^ secretion might occur by activation of the receptor on the epithelial cells

Mueller et al. (2011) [166]	PAR1, PAR2, and PAR4	Human submucosal plexus	Voltage- and calcium-sensitive dye recordings	PAR1, rather than PAR2 and PAR4, activates neurons and glia
		Guinea pig (comparative study)	Voltage- and calcium-sensitive dye recordings	PAR2, rather than PAR1 and PAR4, evoked strong responses in enteric neurons and glia
